# Accelerated epigenetic aging in Werner syndrome

**DOI:** 10.18632/aging.101217

**Published:** 2017-04-04

**Authors:** Anna Maierhofer, Julia Flunkert, Junko Oshima, George M. Martin, Thomas Haaf, Steve Horvath

**Affiliations:** ^1^ Institute of Human Genetics, Julius Maximilians University, Würzburg, Germany; ^2^ Department of Pathology, University of Washington, Seattle, WA 98105, USA; ^3^ Department of Clinical Cell Biology and Medicine, Graduate School of Medicine, Chiba University, Chiba, Japan; ^4^ Department of Human Genetics, David Geffen School of Medicine, University of California Los Angeles, Los Angeles, CA 90095, USA; ^5^ Department of Biostatistics, Fielding School of Public Health, University of California Los Angeles, Los Angeles, CA 90095, USA

**Keywords:** Werner syndrome, progeria, epigenetics, epigenetic clock, DNA methylation

## Abstract

Individuals suffering from Werner syndrome (WS) exhibit many clinical signs of accelerated aging. While the underlying constitutional mutation leads to accelerated rates of DNA damage, it is not yet known whether WS is also associated with an increased epigenetic age according to a DNA methylation based biomarker of aging (the "Epigenetic Clock"). Using whole blood methylation data from 18 WS cases and 18 age matched controls, we find that WS is associated with increased extrinsic epigenetic age acceleration (p=0.0072) and intrinsic epigenetic age acceleration (p=0.04), the latter of which is independent of age-related changes in the composition of peripheral blood cells. A multivariate model analysis reveals that WS is associated with an increase in DNA methylation age (on average 6.4 years, p=0.011) even after adjusting for chronological age, gender, and blood cell counts. Further, WS might be associated with a reduction in naïve CD8+ T cells (p=0.025) according to imputed measures of blood cell counts. Overall, this study shows that WS is associated with an increased epigenetic age of blood cells which is independent of changes in blood cell composition. The extent to which this alteration is a cause or effect of WS disease phenotypes remains unknown.

## INTRODUCTION

Werner syndrome (WS, OMIM: 277700) is an autosomal recessive progeroid syndrome characterized by the appearance of multiple features of aging begin-ning in early adulthood. Approximately 90% of individuals presenting with WS have mutations in the *WRN* gene, which encodes a 1432 amino acid protein with a central domain characteristic of members of the Rec Q family of helicases. The clinical phenotype of WS includes scleroderma-like skin changes, bilateral ocular cataracts, type 2 diabetes mellitus, osteoporosis, hypogonadism, and atherosclerosis. The most common causes of death are cancer and myocardial infarction and the average age at death is 54 years [1, 2].

Little is known about the association of epigenetic alterations with WS. Several recent studies have proposed to measure the physiological age of tissue samples by combining the DNA methylation levels of multiple dinucleotide markers, known as Cytosine phosphate Guanines or CpGs [3-7]. In particular, the “Epigenetic Clock” was developed to measure the age of sorted human cell types (CD4+ T cells or neurons), all tissues, and organs including blood, brain, breast, kidney, liver, and lung [6]. The epigenetic clock is defined as a weighted average across 353 CpG sites. The resulting age estimate (in units of years) is referred to as "DNA methylation age" (DNAm age) or "epigenetic age". Recent studies support the idea that epigenetic age estimates are at least passive biomarkers of biological age. For instance, the epigenetic age of blood has been found to be predictive of all-cause mortality [8-12], frailty [13], lung cancer [14], and cognitive and physical functioning [15]. Further, the utility of the epigenetic clock method using various tissues and organs has been demonstrated in studies of Alzheimer's disease [16], centenarian status [10, 17], Down syndrome [18], HIV infection [19], Huntington's disease [20], obesity [21], lifetime stress [22], menopause [23], osteoarthritis [24], and Parkinson's disease [25]. Despite many diverse applications of the epigenetic clock, we are not aware of any studies that have analyzed epigenetic aging rates in WS.

Here we show for the first time that measures of epigenetic age acceleration are indeed associated with WS status. Different from typical epigenome-wide association studies (EWAS) that interrogate individual CpGs, the current study posits a single hypothesis: WS is associated with epigenetic age acceleration in blood cells. In a secondary analysis, we also relate WS status to abundance measures of blood cell types that were estimated using DNA methylation data.

## RESULTS

### Subjects and tissue

We analyzed DNA methylation levels by the Illumina Infinium MethylationEPIC BeadChip in whole blood of 18 patients with confirmed mutations in the *WRN* gene (16 male, 2 female) and 18 controls, which were matched for age and for gender (with one exception: 15 male, 3 female) (Table 1).

**Table 1:** Sample characteristics of matched WS cases and controls

Sample ID	Disease status	Registry #	gender	age
PWM18	WS	CHAR1010	male	18
CM18	control		male	18
PWM22	WS	KERA1010	male	22
CM22	control		male	22
PWF31	WS	PA1010	female	31
CF31	control		female	31
PWM32	WS	BOERN1010	male	32
CM32	control		male	32
PWF36	WS	TIT1010	female	36
CF36	control		female	36
PWM37-1	WS	AFRI1010	male	37
CM37-1	control		male	37
PWM37-2	WS	TORON1010	male	37
CM37-2	control		male	37
PWM37-3	WS	VELO1010	male	37
CM37-3	control		male	37
PWM38	WS	ZE1010	male	38
CM38	control		male	38
PWM39	WS	MASS1010	male	39
CM39	control		male	39
PWM40	WS	MARY1010	male	40
CM40	control		male	40
PWM43-1	WS	HAWI1010	male	43
CM43-1	control		male	43
PWM43-2	WS	NY1010	male	43
CM43-2	control		male	43
PWM45-1	WS	BIA1010	male	45
CM45-1	control		male	45
PWM45-2	WS	USC1010	male	45
CM45-2	control		male	45
PWM47	WS	CHAP1010	male	47
CM47	control		male	47
PWM49	WS	CONST1010	male	49
CM49	control		male	49
PWF59	WS	TY1010	female	59
CM59	control		male	59

### Accuracy of the epigenetic clock

DNAm age (also referred to as epigenetic age) was calculated as described in [6]. Mathematical details and software tutorials for the epigenetic clock can be found in the Additional files of [6]. An online age calculator can be found at our webpage (https://dnamage.genetics.ucla.edu). All of the described epigenetic measures of aging and age acceleration are implemented in our freely available software.

**Figure 1:**
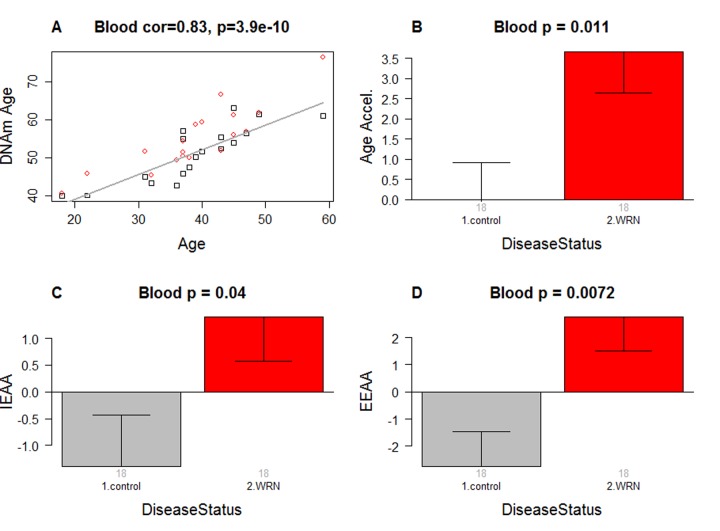
Epigenetic age analysis of Werner syndrome (**A**) DNA methylation age (y-axis) versus chronological age (x-axis). Dots correspond to subjects and are colored by WS status (red=case, black=control). We define three measures of epigenetic age acceleration. (**B**) presents results for the "universal" measure of epigenetic age acceleration, which is defined as residual to a regression line through the control samples, i.e. the vertical distance of a point from the line. By definition, the mean age acceleration in controls is zero. (**C**) The bar plots relate measures of intrinsic epigenetic age acceleration to WS status. This measure is independent of blood cell counts. (**D**) shows findings for the measure of extrinsic epigenetic age acceleration, which does relate to changes in cell composition. Each bar plot depicts the mean value (y-axis), 1 standard error, and the group size (underneath the bar). The p-value results from the Kruskal Wallis test, which is a non-parametric group comparison test.

As expected, DNAm age has a strong linear relationship with chronological age (r=0.83, Figure 1A).

### Werner syndrome is associated with intrinsic and extrinsic epigenetic age acceleration

In this article, we consider three measures of epigenetic age acceleration (Methods). The first measure, which will be referred to as a universal measure of age acceleration (denoted AgeAccel) applies to virtually all tissues and cell types (with the exception of sperm). The other two measures (referred to as intrinsic and extrinsic age acceleration, respectively) only apply to peripheral blood cells. The universal measure AgeAccel is defined as the difference between DNAm age value and the value predicted by a linear regression model in controls. The measure of intrinsic epigenetic age acceleration (IEAA) measures "pure" epigenetic aging effects in blood cells that are not confounded by differences in blood cell counts. The measure of extrinsic epigenetic age acceleration (EEAA) aims to measure aging in immune related components and also relates to age-associated changes in blood cell composition such as the decrease of naïve CD8+ T cells and the increase in memory or exhausted CD8+ T cells [26-28]. EEAA is defined on the basis of a weighted average of the epigenetic age measure from [5] and three blood cell types that are known to change with age: naïve (CD45RA+CCR7+) cytotoxic T cells, exhausted (CD28-CD45RA-) cytotoxic T cells, and plasma B cells. By definition, EEAA has a positive correlation with the amount of exhausted CD8+ T cells and plasma blast cells and a negative correlation with the amount of naïve CD8+ T cells. Blood cell counts were estimated based on DNA methylation data as described in Methods. By construction, our three measures of epigenetic age acceleration are uncorrelated (r=0) with chronological age at the time of blood draw.

WS is significantly associated with epigenetic age acceleration according to all three measures of epigenetic age acceleration (Figure 1B,C,D). The strongest association can be observed for EEAA (p=0.0072).

To estimate the actual amount of age acceleration, we regressed DNAm age on disease status, age, gender, and blood cell count estimates. According to this multi-variate regression model, the blood of WS cases is 6.4 years older than that of age matched controls (Table 2).

**Table 2:** Multivariate model analysis

Covariate	Coef	Std. Error	T-statistic	P-value
Age	0.66293	0.103509	6.4046	6.2×10^−7^
Werner Syndrome	4.250449	1.560597	2.7236	0.011
Gender(female)	−1.97987	2.141069	−0.9247	0.36
CD4+T cell	35.42897	27.09406	1.3076	0.20
Granulocyte	34.03732	19.64958	1.7322	0.094
Natural Killer cell	17.97726	24.01823	0.7484	0.46
Naïve CD8+ T cell	−0.00363	0.021163	−0.1716	0.86

DNA methylation age (outcome) is regressed on chronological age, disease status, gender, and blood cell counts. Note that WS is associated with an increased age of 4.250449/0.662930=6.4 years.

### Conditional logistic regression analysis

Our previous multivariate linear model analysis ignored the fact that cases and controls were grouped into matched pairs. To adjust for this matched pair design, we used a conditional logistic regression analysis that automatically adjusted for chronological age and gender. According to univariate conditional logistic regression models, WS status (dependent variable) is significantly associated with *AgeAccel* (p=0.047, regression coefficient=0.258, standard error=0.130), IEAA (p=0.045, coef=0.271, SE=0.135) and to a lesser extent with EEAA (p=0.071, coef=0.164, SE=0.0907).

### Suggestive evidence for a decreased abundance of naïve CD8+ T cells in Werner syndrome

**Figure 2:**
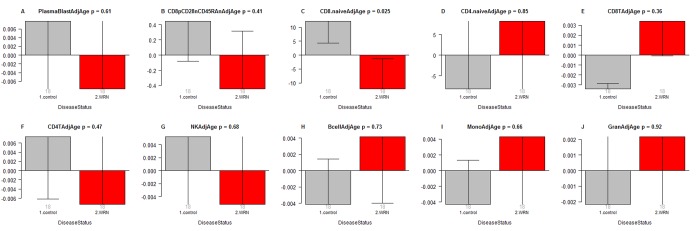
Age adjusted blood cell counts versus Werner syndrome status WS status (x-axis) versus the age adjusted estimate of (**A**) plasma blasts, (**B**) exhausted CD8+ T cells (defined as CD8+CD28-CD45RA-), (**C**) naïve CD8+ T cell count, (D) naïve CD4+ T cell count, (**E**) CD8+ T cells, (**F**) CD4+ T cells, (**G**) natural killer cells, (**H**) B cells, (**I**) monocytes, (**J**) granulocytes. The abundance measures of blood cell counts were estimated based on DNA methylation levels using the epigenetic clock software. Each bar plot depicts the mean value (y-axis), one standard error, and the group size (underneath the bar). The p-value results from a non-parametric group comparison test (Kruskal Wallis).

In a secondary analysis, we related disease status to blood cell count estimates based on DNA methylation data (Figure 2). Comparison of age adjusted blood cell counts between WS patients and controls revealed a significant decrease of naïve CD8+ T cells (CD8+CD45RA + CCR7+) in WS cases (p=0.025, Figure 2C). However, the p-value (p=0.025) is not significant after adjusting for multiple comparisons. Further, WS was not associated with naïve CD8+ T counts in a conditional logistic regression model analysis (p=0.16) that adjusted for the matched pair design. None of the blood cell counts were related to WS status according to our conditional logistic regression model analysis.

## DISCUSSION

Using a novel DNA methylation data set, we demonstrate accelerated epigenetic aging effects in WS. WS has a significant relationship with all three measures of epigenetic age acceleration. The observed accelerated epigenetic aging effects do not reflect confounding due to changes in blood cell composition because a) they can be observed for cell-intrinsic measures of age acceleration (IEAA) and b) they remain significant in multivariate models that adjust for blood cell counts.

Since all of our WS cases had confirmed mutations in the *WRN* gene, the epigenetic aging effects must be a consequence of loss of function mutations in the *WRN* gene. In the following, we discuss several theories that might explain the observed epigenetic age acceleration in blood.

### The helicase theory of accelerated epigenetic aging

The *WRN* gene encodes a 1432 amino acid protein with a central domain characteristic of members of the Rec Q family of helicases. Since the WRN probably plays an important role in a DNA helicase it stands to reason that the observed accelerated epigenetic aging effect in WS results from a process in which DNA helicases play an important role. More generally it might result from a process that involves both DNA and RNA helicases. Helicases are enzymes that bind and may even remodel nucleic acid or nucleic acid protein complexes. There are both RNA helicases and DNA helicases (such as WRN). DNA helicases function in cellular processes where double-stranded DNA must be separated, including DNA replication, DNA repair, and trans-cription. By contrast, RNA helicases are involved in shaping the form of RNA molecules, during all processes involving RNA, such as transcription, splicing, and translation. Interestingly, two recent papers suggest that RNA helicases affect epigenetic aging rates in the cerebellum: a genome-wide association study of epigenetic aging rates in the cerebellum implicated DHX57 (DEAH-Box Helicase 57) which is an RNA helicase [29]. Further, an epigenetic clock analysis of tissues from centenarians found that "helicase activity" might explain the finding that the cerebellum ages more slowly than other brain regions [17]. Overall, these results suggest that both RNA and DNA helicases affect epigenetic aging rates. The helicase theory of epigenetic aging has the following shortcomings. First, the relationship between RNA helicase activity and epigenetic age acceleration could only be observed in the cerebellum (and not yet in peripheral blood cells). Second, DNA helicases (such as WRN) may have little in common with RNA helicases. Third, helicase genes are not a smoking gun for any particular molecular process because they are ubiquitous and essential proteins for many processes.

### The epigenomic instability theory of accelerated epigenetic aging

The WRN protein has exonuclease and helicase activities and is involved in DNA repair, DNA replication, recombination, transcription, and telomere maintenance [30, 31]. Thus, the discovery that loss of function of *WRN* is the cause of WS supports a major role for genomic instability as a fundamental mechanism of aging [32]. A recent study provides evidence of an important role of WRN in the maintenance of chromatin stability of mesenchymal stem cells [40]. We hypothesized that the epigenetic clock might relate to the actions of an epigenomic maintenance system [6]. Under this hypothesis, our results suggest that loss of function of *WRN* affects the epigenomic maintenance system resulting in increased epigenetic age. The main problem with the epigenomic instability theory of accelerated aging is the paucity of mechanistic details.

### The telomere theory of accelerated epigenetic aging

This theory posits that epigenetic age acceleration in WS could result from telomere shortening. It was suggested that genomic instability is caused by telomere loss during DNA replication and that loss of WRN helicase activity promotes telomere loss [33]. Fibroblasts from WS patients grow poorly in culture and display a reduced lifespan. However, ectopic expression of telomerase in WS fibroblast cell lines partially rescues them from accelerated replicative senescence and increases genomic integrity [34, 35]. The average telomere erosion rate in bulk-cultured WS cells was shown to vary from that of normal fibroblasts to four times that of normal. At the single cell level, WS fibroblasts display telomere dynamics not significantly different from those in control fibroblasts, suggesting that the accelerated replicative senescence observed in WS fibroblasts is not caused by accelerated telomere shortening [36]. In contrast, an *in vivo* study showed that TRF (Telomere Restriction Fragment) lengths in skin samples of WS patients in their thirties were comparable with that of healthy samples, but were shorter in older WS patients compared to controls. Regression analyses is showed that the TRF length in skin and muscle of individuals with WS was significantly shorter than those in controls [37]. A major problem with this telomere theory of epigenetic age acceleration is that intrinsic epigenetic age acceleration is not correlated with telomere length in peripheral blood cells or adipose tissue [13, 21, 38].

### The immunosenescence theory of accelerated epigenetic aging

This theory posits that the observed accelerated epigenetic aging effects in blood result from changes in blood cell composition that mirror those observed in immunosenescence. It is well known that a profound age-associated alteration in the T cell compartment is the reduction of naïve CD8+ T cells, which are involved in protection against infectious diseases and play an important role in immune surveillance against malignancies [27]. The theory is supported a) by our suggestive finding regarding the reduction of naïve CD8+ T cells in WS. However, the immunosenescence theory has several severe shortcomings including the following. First, the observed decrease in naïve CD8+ T cells only led to a p-value of 0.025 that is not significant after adjusting for multiple comparisons. This suggestive result requires replication in a larger study preferably involving flow cytometric measures. Second, the theory is inconsistent with our observed lack of an association between exhausted CD8+T cells and WS. Third, the observed epigenetic age acceleration effects are independent of changes in blood cell composition according to our multivariate model analysis.

### Limitations

We acknowledge the following limitations. First, our sample size was relatively small (18 cases and 18 controls). Second, we focused only on peripheral blood cells. Future studies should evaluate whether accelerated epigenetic aging effects can also be found in other tissues. Third, there is an interesting quantitative difference between the degree of accelerated epigenetic aging deduced from these peripheral blood cell studies of WS patients (6.4 years) and the premature ages of death of these patients. The most recently available data from both Japan and the US indicate that the mean age of death for WS patients is 54 years; this contrasts typical longevities in Japan of ∼ 80 years and in the US of ∼74 years [39, 40]. This discrepancy might reflect the fact that the epigenetic age of blood is only an incomplete measure of organismal age. We believe that organismal age will be better estimated by combining the epigenetic age estimates of multiple tissues and organs.

## CONCLUSIONS

We demonstrate that WS is associated with increased epigenetic age acceleration in blood according to several highly robust epigenetic measures of age acceleration. This finding reflects cell-intrinsic epigenetic aging effects that are independent of changes in cell composition. Epigenetic age acceleration of blood is not specific to WS but can also be observed in other conditions such as Down syndrome [18] and to a lesser extent in Parkinson's disease [25]. The degree to which these observed epigenetic age acceleration effects are causes or effects of the many geriatric disorders seen in WS remains unknown. A recent study providing evidence of an important role of WRN in the maintenance of chromatin stability of mesenchymal stem cells, however, is consistent with an important causal role [41].

## MATERIALS AND METHODS

### Methylation analysis

Whole blood DNA samples of WS patients were sent to us from the International Registry of Werner Syndrome (http://www.wernersyndrome.org/; Seattle, WA). Whole blood samples of controls were collected in the Institute of Human Genetics of the University of Wuerzburg. Sodium bisulfite conversion of genomic DNA was performed using the EZ DNA Methylation™ Kit (Zymo Research, Irvine, CA, USA) according to the provided manual. The Infinium MethylationEPIC BeadChip (Illumina, San Diego, CA, USA) was used according to the manufacturer's protocol to analyze genome-wide DNA methylation. Chips were scanned by iScan (Illumina). Genome Studio (Illumina) was used for background subtraction and normalization to internal controls.

### Measures of epigenetic age acceleration

The name of our universal measure of age acceleration (*AgeAccel*) reflects that it applies to virtually all sources of human DNA (with the exception of sperm). Here we defined it as follows. First, we regressed DNAm age on chronological age in controls. Next, we used the resulting model to predict the DNAm age of each subject. Next, the universal measure was defined as the difference between the observed measure of DNAm age and the predicted value. Thus, a high positive value for *AgeAccel* indicates that the observed DNAm age is higher than that predicted based on controls. *AgeAccel* has a relatively weak correlation with blood cell counts [19] but it still relates to blood cell counts.

To subtract out the effect of blood cell counts, we find it useful to define a measure of intrinsic epigenetic age acceleration (IEAA) which measures "pure" epigenetic aging effects that are not confounded by differences in blood cell counts. It is defined as the residual resulting from a multivariate regression model of DNAm age on chronological age and various blood immune cell counts (naïve CD8+ T cells, exhausted CD8+ T cells, plasma B cells, CD4 T cells, natural killer cells, monocytes, and granulocytes).

The measure of extrinsic epigenetic age acceleration (EEAA) aims to measure epigenetic aging in immune related components. EEAA is defined using the following three steps. First, we calculated the epigenetic age measure from Hannum et al based on 71 CpGs [5]. The resulting age estimate is correlated with certain blood cell types [9]. Second, we increased the contribution of blood cell types to the age estimate by forming a weighted average of the Hannum estimate with 3 cell types that are known to change with age: naïve (CD45RA+CCR7+) cytotoxic T cells, exhausted (CD28-CD45RA-) cytotoxic T cells, and plasma B cells using the approach of [42]. The resulting measure of blood age is referred to as BioAge4 in our epigenetic clock software. Third, we defined a measure of age acceleration (EEAA) as the residual resulting from a univariate model regressing BioAge4 on chronological age. By definition, our measure of EEAA has a positive correlation with the amount of exhausted CD8+ T cells and plasma blast cells and a negative correlation with the amount of naïve CD8+ T cells. EEAA is known as BioAge4HAStaticAdjAge in our software.

Blood cell counts were estimated based on DNA methylation data as described in the section entitled "Estimating blood cell counts based on DNA methylation levels". By construction, EEAA tracks both age-related changes in blood cell composition and intrinsic epigenetic changes. By definition, none of our three measures of epigenetic age acceleration are correlated with the chronological age. IEAA differs across ethnic groups [43] but has a negligible association with behavioral/lifestyle factors [44].

### Estimating blood cell counts based on DNA methylation levels

We estimate blood cell proportions using two different software tools. Houseman's estimation method [45], which is based on DNA methylation signatures from purified leukocyte samples, was used to estimate the proportions of CD8+ T cells, CD4+ T, natural killer, B cells, and granulocytes. Granulocytes are also known as polymorphonuclear leukocytes. The advanced analysis option of the epigenetic clock software [6, 19] was used to estimate the percentage of exhausted CD8+ T cells (defined as CD28-CD45RA-) and the number (count) of naïve CD8+ T cells (defined as (CD45RA+CCR7+). Using another data set, we found that estimated blood cell counts correlate strongly with corresponding flow cytometric measurements r = 0.63 for CD8 + T cells, r = 0.77 for CD4+ T, r = 0.67 B cell, r = 0.68 naïve CD8+ T cell, r = 0.86 for naïve CD4+ T, and r = 0.49 for exhausted CD8+ T cells [43].
